# On the joy of interdisciplinary research and field work: Constanze Kuhlisch navigating her career in marine microbiology

**DOI:** 10.1038/s42003-024-06532-8

**Published:** 2024-07-11

## Abstract

Conducting research at the interface of chemistry, microbiology and marine biology, Constanze Kuhlisch reports on challenges in science due to COVID and the situation in Israel, while preparing grants to start her own research group.

Dr. Constanze Kuhlisch is a research associate in the laboratory of Assaf Vardi at the Weizmann Institute in Israel. Since her time as a PhD student in the group of Georg Pohnert in Jena, Germany, she applies environmental metabolomics approaches to study host-pathogen interactions in the ocean. She is currently researching tripartite algae-virus-bacteria interactions and their consequences for the marine metabolic landscape, a project funded by the Minerva Stiftung, which supports cooperative German-Israeli research. At present, she is applying for grants to start her own research.

**Figure:**
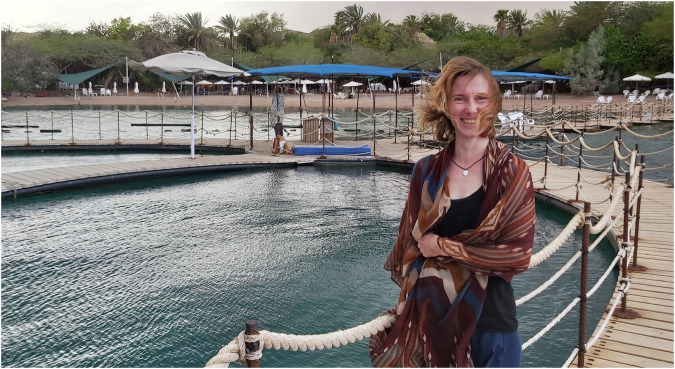
Martin Sperfeld

After you conducted your PhD work in Jena, Germany, what led you to the Weizmann Institute in Israel?

Jena is a real hub for chemical ecology. After submersing myself during the PhD into analytical chemistry and emerging metabolomics tools, I wanted to bring my analytical chemistry expertise back to marine ecology research. My aim was to join a lab that investigates a model system, and in which students naturally work together, to push the boundaries of knowledge synergistically by combining their individual skills and interests.

Then I saw a call from Assaf Vardi to join his lab at the Weizmann Institute of Science. It seemed perfect: they study the cosmopolitan microalga *Emiliania huxleyi* and combine mechanistic research in the lab with field work to reveal the ecological impact of its blooms. One two-hour Skype call later, which passed in no time, and I sat in a plane to visit the lab, the institute, and the country… and eventually join the lab for my postdoc and thrive in a place that fosters curiosity and fundamental research.

Your work sits at the intersection of biology and chemistry – would you say you belong to one subject more than the other (e.g. at conferences or when submitting to journals) or do such artificial borders not apply to your work at all?

I might be in favor of ‘interface’ rather than ‘border’ to describe the field of chemical ecology. There is so much beautiful life because of chemical reactions, and there are so many cool metabolites because of cells directing their formation. In my work, I need to merge both fields. The consequence of studying biology for five years is, however, that I am more familiar with the language and concepts of marine ecology. And I am more passionate about, for instance, reconstructing the physiological challenges of a five micrometer-sized, non-motile, sun-loving microalga during midsummer in the North Atlantic than about reconstructing the dynamic changes in the electron distribution around a molecule upon nucleophilic attack.

From my experience, I face similar communication problems in this interdisciplinary research as during traveling. Each research area has its own vocabulary, and this crux must be overcome for a fruitful scientific exchange. There is a society for chemical ecology (ISCE), but ultimately, I often choose between more biology- or chemistry-oriented communities and journals. As a chemist, I really enjoyed the Gordon Research Conference (GRC) Marine Natural Products, while at the GRC Marine Microbes I enjoyed great ecological discussions, and the Ringberg Symposium is the perfect community to talk about giant viruses.

The COVID pandemic happened right in the middle of your postdoctoral research time. How was the situation in Israel and how did it affect your scientific work? How does the current, challenging situation in Israel affect you?

Israel was very quick in having an infrastructure to get vaccinated. On the other hand, we had high infection numbers leading to several lockdowns, some of them with a 100-meter restriction for leaving home. The only way to get in and out of Israel is by flying, and the regulations that existed around entering the country and going into quarantine on campus left some students abroad for months and made me not leave the country for over a year. Luckily, the campus was never completely inaccessible. The capacity on campus was restricted and we had a shift system, but I could go to the lab almost every day for a few hours. Most social interactions were online, which for an international student in a foreign country can be tough. But there were positive effects as well! During the first lockdown, I started writing a manuscript, and we organized joined lab meetings with other labs and researchers.

The recent terror attack changed my life and work much more profoundly than COVID. It was as if someone pressed a ‘stop’ button, and I found myself cutting vegetables in a restaurant in Tel Aviv to supply the suddenly recruited soldiers, including many of my colleagues and friends. The labs on campus were completely deserted. More than 60% of the international students left the campus within the first two weeks, and many Israeli colleagues either were recruited or had to watch the kids at home as there were no kindergartens or schools. It took me some time to adapt to this reality. And now we are facing the political consequences of the ongoing war. It effects the freedom of intellectual exchange between scientists and creates invisible borders between otherwise enthusiastic marine ecologists.

You started to write grant applications as an early-career independent researcher. How did this turn out and which tips could you give younger postdocs with similar goals?

My first grant application was actually a travel grant during my bachelor’s degree to fly to Australia and study the pollination of *Utricularia menziesii*, a small carnivorous plant that grows on rock outcrops and is reported to be pollinated by birds. During the last years as a postdoc, I was involved in grant writing for larger research projects, fellowships and to get a new mass spectrometer for the lab. Some of them were successful, some of them not. In all cases, it was good to start early on to have enough time to read, discuss, rethink, and compensate for days in which sentences are formed in slow-motion. Besides the timing, it is good to get feedback from colleagues during the writing process, but also to study thoroughly the reviewers’ comments if its rejected. And for me, the place matters - I tried many desks in different places to find a good writing environment that kept me focused and concentrated and let my mind drift away when needed. My favorite spot is in a small library in company with other researchers and with windows all around watching old, wrinkled ficus trees.

If you could choose freely, where would you like to work?

If the Tara Ocean Foundation and its schooner were an institute, I would apply immediately!

In December 2021, I participated in the European AtlantECO project ‘Mission Microbiome’ and sailed for three weeks from Buenos Aires to Ushuaia, a small town in the South of Argentina appropriately termed ‘fin del mundo’, end of the world. We hunted blooms of *Emiliania huxleyi*, which occur every year during Austral summer along the Patagonian shelf break. With the Tara schooner, we followed a bloom patch for several days by drifting with the currents to study microbial interactions in real time in the natural environment. For three weeks, the 36-m sailing boat was defining the borders of my life in the open ocean. It is a fascinating workplace – people from around the world come together to study the ocean in a mutual collaborative effort, including researchers, sailors, and the cook. But there is also a journalist and an artist onboard to let the society participate in this journey. On that note: why not have artists join and rotate through research institutes?

At the moment you frequently do field work; is this something you want to do your entire career?

Yes, surely! As human beings, we are tightly entangled with the environment that surrounds us. We introduced per- and polyfluoroalkyl substances (PFAS) into the industrial world. From there, they entered rivers and groundwater, and now are part of the water cycle and are raining back on us. Similarly, but naturally, the activity of marine microorganisms has effects on global processes like cloud formation or carbon export. We need to better understand the environment to coexist and coevolve on the long term. And that’s what field work is good for.

When incorporating field work in my research, I have the possibility to identify and reveal the daily routines of nature that evolved over long time scales to sustain life in the ocean, but that are silenced under the rigid conditions that we provide in the laboratory. Field work also reminds me of my fascination for nature that once motivated me to study biology. And I really enjoy the social experience of being part of one collective effort to conduct research in a group of people that live and work together 24/7 for several weeks with all its accompanying intensity and challenges. But field work shouldn’t be done just for research’s sake and can benefit from a better infrastructure to analyze and publish the collected data.

*This interview was conducted by associate editor Tobias Goris*

